# The PTX3/TLR4 autocrine loop as a novel therapeutic target in triple negative breast cancer

**DOI:** 10.1186/s40164-023-00441-y

**Published:** 2023-09-25

**Authors:** Arianna Giacomini, Marta Turati, Elisabetta Grillo, Sara Rezzola, Gaia Cristina Ghedini, Ander Churruca Schuind, Eleonora Foglio, Federica Maccarinelli, Jessica Faletti, Serena Filiberti, Angela Chambery, Mariangela Valletta, Laura Melocchi, Stephanie Gofflot, Barbara Chiavarina, Andrei Turtoi, Marco Presta, Roberto Ronca

**Affiliations:** 1 Department of Molecular and Translational Medicine, University of Brescia, Brescia, Italy; 2 Department of Environmental, Biological and Pharmaceutical Sciences and Technologies (DiSTABiF), University of Campania ’Luigi Vanvitelli’, Caserta, Italy; 3 Pathology Unit, Fondazione Poliambulanza Hospital Institute, Brescia, 25121 Italy; 4 BIOTHEQUE, University of Liege, Liege, Belgium; 5 Institut de Recherche en Cancérologie de Montpellier, INSERM U1194, University of Montpellier, Montpellier, France

**Keywords:** PTX3, Triple negative breast cancer (TNBC), TLR4 signaling pathway

## Abstract

**Background:**

The pattern recognition receptor long pentraxin-3 (PTX3) plays conflicting roles in cancer by acting as an oncosuppressor or as a pro-tumor mediator depending on tumor context. Triple negative breast cancer (TNBC) represents the most aggressive histotype of breast cancer, characterized by the lack of efficacious therapeutic targets/approaches and poor prognosis. Thus, the characterization of new molecular pathways and/or alternative druggable targets is of great interest in TNBC.

**Methods:**

The expression of *PTX3* in BC tumor samples and in BC cell lines has been analyzed using the Gene Expression-Based Outcome for Breast Cancer Online (GOBO), qPCR, Western blot and ELISA assay. The contribution of tumor and stromal cells to PTX3 production in TNBC was assessed by analyzing single cell RNA sequencing data and RNAscope performed on TNBC tumor samples. In order to investigate the effects of *PTX3* in TNBC, different cell lines were engineered to knock-down (MDA-MB-231 and BT549 cells) or overexpress (MDA-MB-468 and E0771 cells) PTX3. Finally, using these engineered cells, in vitro (including gene expression profiling and gene set enrichment analyses) and in vivo (orthotopic tumor models in immune-compromised and immune competent mice) analyses were performed to assess the role and the molecular mechanism(s) exerted by PTX3 in TNBC.

**Results:**

*In silico* and experimental data indicate that PTX3 is mainly produced by tumor cells in TNBC and that its expression levels correlate with tumor stage. Accordingly, gene expression and in vitro results demonstrate that PTX3 overexpression confers a high aggressive/proliferative phenotype and fosters stem-like features in TNBC cells. Also, PTX3 expression induces a more tumorigenic potential when TNBC cells are grafted orthotopically in vivo. Conversely, PTX3 downregulation results in a less aggressive behavior of TNBC cells. Mechanistically, our data reveal that PTX3 drives the activation of the pro-tumorigenic Toll-like receptor 4 (TLR4) signaling pathway in TNBC, demonstrating for the first time that the PTX3/TLR4 autocrine stimulation loop contributes to TNBC aggressiveness and that TLR4 inhibition significantly impacts the growth of PTX3-producing TNBC cells.

**Conclusion:**

Altogether, these data shed light on the role of tumor-produced PTX3 in TNBC and uncover the importance of the PTX3/TLR4 axis for therapeutic and prognostic exploitation in TNBC.

**Graphical abstract:**

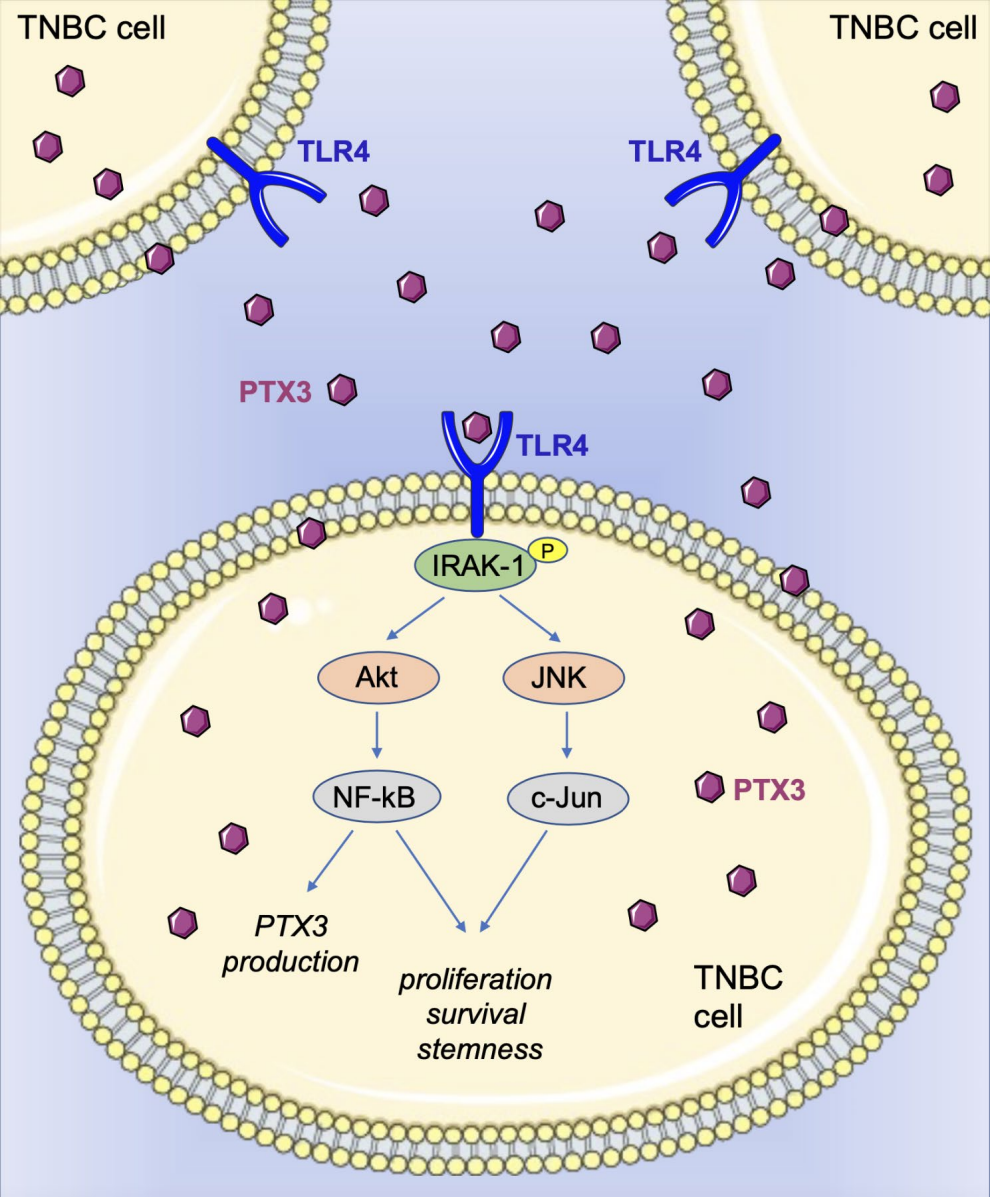

**Supplementary Information:**

The online version contains supplementary material available at 10.1186/s40164-023-00441-y.

## Background

With over 2 million new cases in 2020, breast cancer (BC) is the most common cancer occurring in women and the first most common type of tumor overall (source World Cancer Research Fund). BC represents a heterogeneous disease classified in several complex subsets on the basis of cellular compositions, molecular alterations, and clinical behavior.

Molecular subtyping of BC is now based on classical immunohistochemistry markers such as estrogen receptor (ER), progesterone receptor (PR), and human epidermal growth factor receptor 2 (HER2) that led to the distinction between luminal (A and B), basal, and HER2-positive classes [1, 2]. Luminal A are the most prevalent type of BC (58.5%) and are ER^+^/PR^+^/HER2^−^ and Ki-67^low^; Luminal B account for around 14% of BC and are ER^+^/PR^+^/HER2^−^ and Ki-67^high^; HER2-positive BC represent 11.5% of BC and are ER^−^/PR^−^/HER2^+^. Finally, the 15–20% of BCs are basal-like and are referred as triple negative breast cancers (TNBCs) due to the absence of classical molecular markers (ER^−^/PR^−^/HER2^−^) [3, 4]. Due to its aggressive biological behavior and the lack of potential markers and targets, TNBC represents the most dangerous BC subtype, with the poorest prognosis and outcome [5].

The soluble pattern recognition receptor Long Pentraxin-3 (PTX3) is a member of the pentraxin family and is produced locally in response to inflammatory signals as a functional component of the innate immunity. PTX3 exerts non-redundant functions in various physio-pathological conditions and it has been described to be involved in tumor cell proliferation, angiogenesis, metastatic dissemination and cancer immune-modulation [6–8]. As a secreted protein, PTX3 can be produced and released by both tumor and stroma cells, depending on tumor type [9]. Different studies reported the role of PTX3 as an oncosuppressor acting through the modulation of tumor-associated inflammation [10] and/or by blocking pro-tumor growth factors like various members of the FGF family [9, 11]. Indeed, tumor and/or host PTX3 overexpression can inhibit FGF-driven epithelial-to-mesenchymal transition (EMT) and tumor/metastatic burden in melanoma models [12] and hampers cancer growth in models of fibrosarcoma, prostate and bladder cancer [6, 8, 13]. On the other hand, PTX3 has been shown to promote cell migration and invasion in some experimental tumor models, its expression levels being correlated with tumor progression in different human tumor types. For instance, high levels of PTX3 have been reported in all subtypes of human soft tissue liposarcoma [14] as well as in pancreatic carcinoma cells and in advanced gastric cancer tissues where it promotes the migratory potential of tumor cells and macrophages recruitment [15, 16]. Similarly, PTX3 levels in cervical cancers and gliomas appear to correlate with tumor grade and severity in vitro and in patients [17, 18]. However, even though the mechanisms by which PTX3 exerts its anti-tumor activity are at least partially known, the mechanisms by which PTX3 exerts its tumorigenic activity have still to be revealed.

Recent studies suggest a possible tumorigenic role of PTX3 also in BC. Indeed, high expression levels of *PTX3* have been found to be associated with EMT in high-grade ductal infiltrating carcinomas [19]. Elevated expression of *PTX3* has been observed in distant bone metastases of BC and correlated with osteoclast formation, suggesting that PTX3 might be involved also in the osteolytic bone metastatic process in BC [20]. In addition, *PTX3* expression has been shown to be regulated by PI3K and to foster tumor stem-like features and bad prognosis in basal-like TNBC [21, 22]. However, several issues still remain to be addressed in order to clarify (i) if PTX3 is differentially expressed by the different BC subtypes, (ii) which is the main source of PTX3 (tumor or stromal cells), and (iii) what are the biological effects and the molecular mechanism(s) exerted by PTX3 in BC.

In this study we show that, if compared to the other BC subtypes, high levels of PTX3 are mainly found in TNBC, where *PTX3* transcript is predominantly expressed by tumor cells in respect to cells associated with tumor stroma/microenvironment. Also, in vitro and in vivo data show that PTX3 confers more aggressive biological features to TNBC cells, resulting in augmented tumor cell proliferation and growth. Importantly, we demonstrate that the pro-tumor activity of PTX3 is exerted *via* the activation of the TLR4 pathway which is known to play a relevant role in TNBC aggressiveness [23, 24]. Indeed, our findings reveal for the first time that TNBC cell aggressiveness is fostered by a PTX3/TLR4 autocrine loop of stimulation, and that its inhibition may represent a promising therapeutic approach for the treatment of the most dangerous BC subtype.

## Materials and methods

### Reagents and cell cultures

The TLR4 inhibitor TAK-242 was purchase from Selleckchem (Houston, TX, USA). Human MDA-MB-231, MDA-MB-468 cells were obtained from American Type Culture Collection (ATCC) and cultured in DMEM *plus* 10% FBS; BT549 cells were obtained from American Type Culture Collection (ATCC) and cultured in RPMI *plus* 10% FBS and 1 µg/mL of bovine insulin; murine E0771 cells, derived from a spontaneous mammary tumour in a C57BL/6 mouse were kindly provided by R. Giavazzi (Istituto M. Negri, Milan, Italy) and cultured in DMEM *plus* 20% FBS [25].

For overexpression, breast cancer cells were infected with a pLentiPGK-Puro (Addgene Plasmid #19,070) lentiviral vector harbouring or not the full length human PTX3 cDNA (GenBank accession n° X63613). For silencing, cells were infected with lentiviral vector containing short-hairpin RNA (shRNA) targeting human PTX3 (TRCN0000436981 or TRCN0000430959) or a non-targeting/control sequence (SHC002V, Merck Millipore, Burlington, MA, USA). Transduced cells were selected with 1 µg/ml puromycin. Cells were authenticated by microsatellite genotyping before the starting of the project and periodically all along the project, maintained at low passage, returning to original frozen stocks every 3 to 4 months, and tested regularly for Mycoplasma negativity by PCR and DAPI staining.

### Analyses on human samples

Breast cancer samples used for Western blot, RNAscope analyses and single cell RNA-seq were from different source. Western blot samples were from the institutional biobank of the University Hospital Liege Belgium and clinical data available are reported in Table S1. TNBC samples used for RNAscope (cases #A-D) were from the Unit of Pathology (Spedali civili di Brescia, Italy). TNBC cases analysed in single cell RNA-seq are derived from the publication [26] (see details below).

### Single cell RNA-seq analysis

To examine PTX3 expression in breast cancer we reanalysed previously published single cell RNA-seq analysis involving 5 TNBC patients [26]. The selected cases were all TNBC tumors with no pre-treatment, fulfilling the following histological criteria: staining by immunohistochemistry for estrogen receptor (< 1%) and progesterone receptor (< 1%), and fluorescence in situ hybridization analysis of HER2 amplification using the CEP-17 centromere control probe (ratio of HER2/CEP-17 < 2.2). Processed 10X Genomics (Pleasanton, CA, USA) data were downloaded from GEO repository (GSE148673). The data were imported into R computational environment (4.0) and then analysed using *Seurat 3.1* package using default parameters [27].

### In vitro assays

*Cell Proliferation.* Cells were seeded (10^4^) in 48-well culture plates in complete medium, detached at different time points and counted using the MACSQuant Analyzer (Miltenyi Biotec, Bergisch Gladbach, Germany).

*Clonogenic Assay.* Five hundred cells were seeded in 6-well culture plates and incubated in complete growth medium until visible colonies were formed. Then, the supernatant was removed and cells stained with 0.1% crystal violet/20% methanol. Plates were photographed to count formed colonies using the ImageJ software. Finally, crystal violet staining was solubilized with 1% SDS solution to measure absorbance at 595 nm.

*Soft Agar Assay.* Cells (5 × 10^4^) were suspended in 3ml of complete growth medium containing 0.3% agar and poured on to 2ml pre-solidified 0.6% agar in a 6-well plate. After 3 weeks of incubation, colonies were observed under a phase contrast microscope, photographed, and their area was measured using the ImageJ Software and the SA_NJ algorithm [8].

*Wound-Healing assay.* Confluent cells were scraped with a 200 µl tip to obtain a 2-mm-thick denuded area. After 24 h, wounded monolayers were photographed and the width of the wounds was measured in 3-independent sites per group.

### qPCR analysis

Total RNA was extracted using QIAzol reagent, treated with DNAse and 2 µg of total RNA were retro transcribed with MMLV-RT using random hexaprimers, cDNA was analyzed by quantitative PCR using primers specific for human or murine PTX3 (hPTX3: Forward primer: 5’-CATCTCCTTGCGATTCTGTTTTG-3’; reverse primer: 5’-CCCATTCCGAGTGCTCCTGA-3’). Housekeeping gene human GAPDH was detected for normalization (hGAPDH: Forward primer: 5’-GAAGGTCGGAGTCAACGGATT-3’; reverse primer: 5’-TGACGGTGCCATGGAATTTG-3’).

### Western blot analysis

Cells and fresh frozen tumor tissues were homogenized in NP-40 lysis buffer (1% NP-40, 20 mM Tris–HCl pH 8, 137 mM NaCl, 10% glycerol, 2 mM EDTA, 1 mM sodium orthovanadate, 10 µg/mL aprotinin, 10 µg/mL leupeptin). Protein concentrations were determined using the Bradford protein assay (Bio-Rad Laboratories, Milano, Italy). Then, 30 µg protein/sample were separated by SDS-PAGE and blotted on a PVDF membrane. The following antibodies were used: anti-PTX3 (from B. Bottazzi, Humanitas Clinical Institute, Rozzano, Italy), anti-TLR4 (Bio-Rad), anti-phospho IRAK1 (Sigma-Aldrich, MO, USA), anti-phospho AKTser473 (Cell Signaling Technology, MA, USA), anti-phospho p65 (Santa Cruz Biotechnology, CA, USA). To normalize the amount of loaded proteins, all blots were probed with anti-β-actin (Sigma-Aldrich), anti-α-tubulin (Sigma-Aldrich), anti-GAPDH (Santa Cruz Biotechnology) or anti-HSC70 (Santa Cruz Biotechnology) antibodies. All primary antibodies were diluted 1:1000 and the secondary HRP-conjugated antibodies 1:5000. Chemiluminescent signal was automatically acquired by ChemiDoc™ Imaging System (Bio-Rad) at a final resolution of 62.2 pixel/mm^2^.

### Genome-wide expression profiling (GEP)

GEP was performed on shNT/shPTX3 MDA-MB-231 and mock/PTX3 MDA-MB-468 cells. Total RNA was extracted using TRIzol Reagent according to manufacturer’s instructions (Invitrogen, Waltham, MA, USA). RNA integrity and the purity of the treated cells were assessed using a Bioanalyzer (Agilent Technologies, Santa Clara, CA, USA). Hybridization to HuGene-2_1-st-v1 array strips (ThermoFisher Scientific, Waltham, MA, USA) was performed. Normalized data were imported into Partek® Genomic Suite® 6.6 software (Partek, Chesterfield, MO, USA). After quality controls, Analysis of variance (ANOVA) test was performed to assess the effects of PTX3 modulation on gene expression, comparing MDA-MB-231 shNT vs. MDA-MB-231 shPTX3 and MDA-MB-468 mock vs. MDA-MB-468 PTX3. A cut-off of p-value < 0.01 (FDR corrected) and Log2 fold change ± 2 was applied to select differentially expressed genes. Specific cellular pathways and biological networks modulated by differentially expressed genes were identified through the Core Analysis function in Ingenuity Pathway Analysis (IPA) software (QIAGEN, Hilden, Germany). To identify significantly enriched or depleted groups of genes involved in the same biological pathways, Gene Set Enrichment Analysis (GSEA) on GEP data was performed (http://software.broadinstitute.org/gsea/index.jsp).

### Tumor sphere formation assay and ALDH analysis

Five thousand cells were resuspended in DMEM/F-12 medium (GIBCO) containing 10 ng/ml basic Fibroblast Growth Factor (bFGF), 10 ng/ml Epidermal Growth Factor (EGF) and 2% of B27 supplement (Sigma-Aldrich) and plated into each well of 24-well Ultra-Low Attachment Plates (Corning, NY, USA). After 7 days of incubation, tumor spheres were counted and assayed for ALDH activity using the Aldefluor kit (Stemcell technologies, Vancouver, Canada) according to manufacturer’s instructions. ALDH-positive cell was quantified by cytofluorimetric analysis (MACSQuant Analyzer). Samples treated with the specific ALDH inhibitor diethylaminobenzaldehyde (DEAB) were used as controls to set the gates defining the ALDH-negative and the ALDH-positive regions.

### Targeted quantitative analysis of secreted cytokines by Bio-Plex assay

The targeted quantitative analysis of secreted cytokines and chemokines in culture media was performed by using the Bio-Plex multiplex system (Bio-Rad) based on xMAP technology [28]. Magnetic beads labeled with red and infrared fluorophores are coated with specific antibodies, thus allowing the simultaneous detection of multiple target analytes within one sample. Following reaction of beads with target analytes, detection is performed with a biotinylated antibody and phycoerythrin conjugated streptavidin. All steps were performed according to manufacturer’s instructions. Data were acquired using a Bio-Plex MAGPIX Multiplex Reader system (Bio-Rad).

### In vivo studies

Animal Experiments were performed according to the Italian laws (D.L. 116/92 and following additions) that enforce the EU 86/109 Directive and were approved by the local animal ethics committee (OPBA, Organismo Preposto al Benessere degli Animali, Università degli Studi di Brescia, Italy).

Seven-week-old NOD/Scid female mice were injected orthotopically into the mammary fat pad with 4 × 10^6^ MDA-MB-231 (shNT or shPTX3) and 8 × 10^6^ MDA-MB-468 (mock or PTX3), while seven-week-old syngeneic C57BL/6 females were injected orthotopically with 5 × 10^5^ E0771 (mock or PTX3) cells.

TAK-242 treatment (3 mg/Kg) was performed IP every other day when tumors were palpable. Tumors were measured with callipers and the volume was calculated according to the formula V = (D × d2)/2, where D and d are the major and minor perpendicular tumor diameters, respectively. At the end of the experimental procedure, tumors were surgically removed, weighed and paraffin embedded for immunohistochemical analysis.

### Immunohistochemical and RNAscope analyses

For IHC on tumor xenograft samples, formalin-fixed, paraffin-embedded samples were sectioned at a thickness of 3 μm, dewaxed, hydrated, and stained with hematoxylin and eosin (H&E) or processed for immunohistochemistry with rabbit anti-human PTX3 (from B. Bottazzi, Humanitas Clinical Institute, Rozzano, Italy), rabbit anti-human phospho-Histone H3 (Merck Millipore), rabbit anti-CD44 (ThermoFisher Scientific), rabbit anti-phospho IRAK1 (Sigma-Aldrich) or rabbit anti-phospho p65 (Santa Cruz Biotechnology) antibodies. Positive signal was revealed by 3,3’-diaminibenzidine (Roche) stainings. Sections were finally counterstained with Carazzi’s hematoxylin before analysis by light microscopy. Images were acquired with the automatic high-resolution scanner Aperio System (Leica Biosystems, Wetzlar, Germany, EU) and image analysis was carried out using the open-source ImageJ software.

For RNAscope on TNBC patients’ samples, in situ hybridization was performed on FFPE TNBC biopsies using RNAscope® 2.5 HD Reagent Kit (RED 322,360, Advanced Cell Diagnostics (ACD), Hayward, CA). Sections were heated at 60 °C for 1 h and deparaffinized in fresh xylene. After dehydration in 100% ethanol, sections were incubated with the H_2_O_2_ for 10 min, target retrieval reagent for 15 min, and protease for 30 min (Pretreatment kit 322,330, ACD). The sections were then covered with a probe Hs-PTX3 (ref. 517,611) in the HybEZ oven (ACD) at 40 °C for 2 h. The Hs-PPIB probe was used as a control to ensure RNA quality. After probes’ hybridizations, sections were subjected to signal amplification using the HD 2.5 detection Kit, and hybridization signal was detected using a Fast- RED solution. Breast cancer biopsies were obtained from the institutional biobank of the University Hospital Liege Belgium, following the approval of the institutional ethical committee (reference number 2009/69).

### Statistical analyses

Statistical analyses were performed using Prism 8 (GraphPad Software). Student’s *t* test for unpaired data (2-tailed) was used to test the probability of significant differences between two groups of samples. For more than two groups of samples, data were analyzed with a 1-way analysis of variance and corrected by the Bonferroni multiple comparison test. Tumor volume data were analyzed with a 2-way analysis of variance and corrected by the Bonferroni test. Differences were considered significant when *p* < 0.05 unless otherwise specified.

## Results

### PTX3 is highly expressed in TNBC cells

**Fig. 1:**
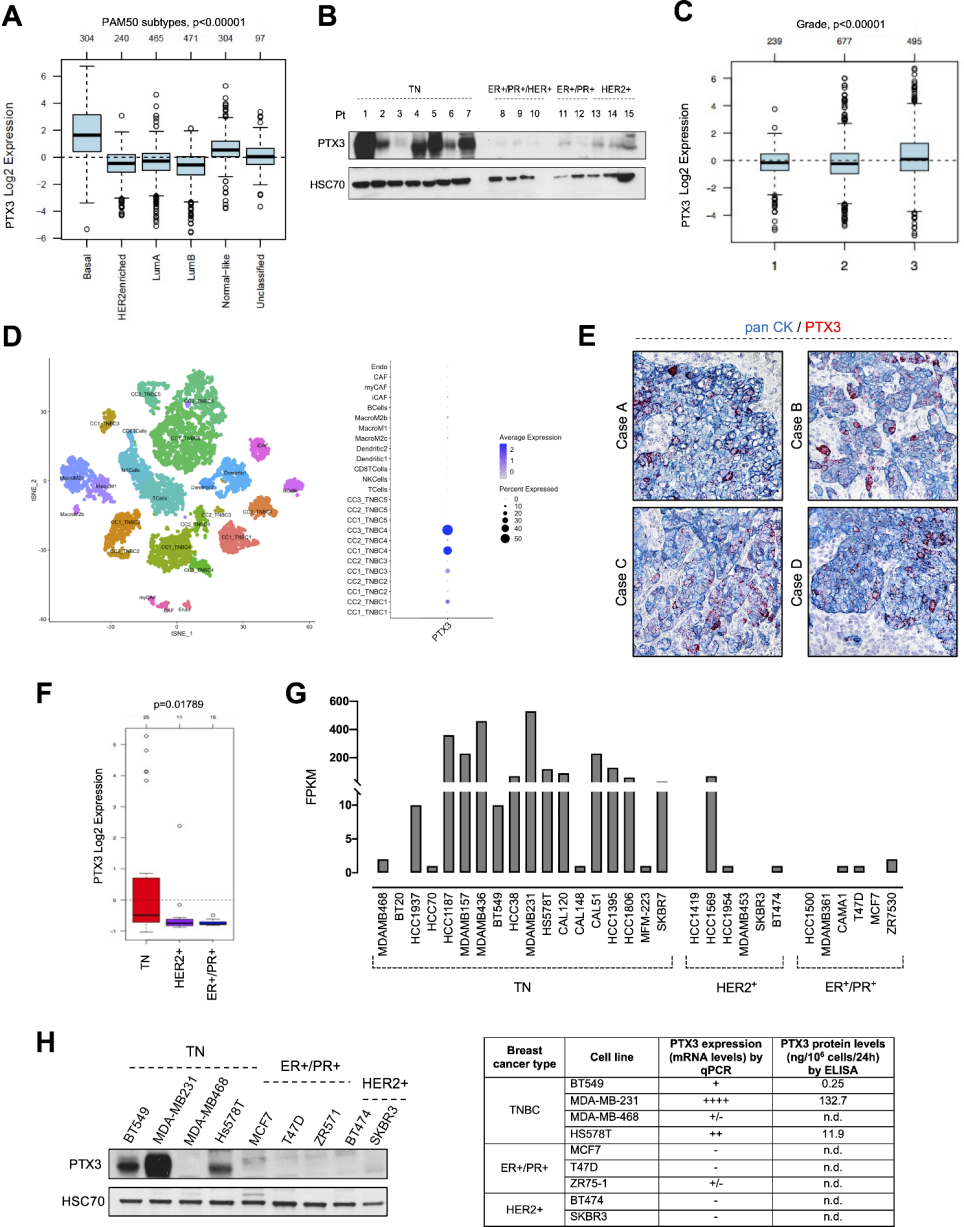
PTX3 expression in human BC. (**A**) PTX3 mRNA expression in patient-derived tumor samples by GOBO database. (**B**) Western blot analysis of PTX3 expression in patient-derived tumor samples. (**C**) Correlation of PTX3 expression with tumor grade by GOBO database. **D**) Single cell analyses of TNBC samples (left panel) and PTX3 expression in each cell population (right panel). (**E**) RNAscope analysis for PTX3 expression (red staining) in patient-derived tumor samples. Tumor cells are identified by immunohistochemical staining for pan cytokeratin (CK) expression (blue membrane staining). (**F**) Data from GOBO database about *PTX3* expression in BC cell lines at mRNA level. (**G**) Data from EMBL-EBI expression atlas database about *PTX3* expression in BC cell lines at mRNA level. FPKM: Fragments Per Kilobase of transcript per Million map reads. (**H**) PTX3 expression by qPCR and Western blot analyses and PTX3 secreted levels by ELISA from several BC cell lines

The expression of *PTX3* has been analyzed in tumor samples from BC patients using the Gene Expression-Based Outcome for Breast Cancer Online (GOBO) [29, 30]. As shown in Fig. 1A, *PTX3* expression is significantly more elevated in basal-like TNBC than in all other types of BCs. Accordingly, Western blot analysis performed on samples from triple negative (TN), triple positive, ER^+^/PR^+^ and HER2^+^ tumor biopsies (Table S1) confirmed the prevalent expression of PTX3 in basal-like TNBC (Fig. 1B). Interestingly, in line with a described negative prognostic correlation between PTX3 expression and overall survival in TNBC [21], the levels of *PTX3* mRNA correlate with BC grading (Fig. 1C).

In order to understand the relative contribution of tumor and stromal/immune cells to PTX3 production in TNBC, analysis of single cell RNA sequencing data was performed on tumor samples obtained from TNBC patients (Fig. 1D) [26]. As shown in Fig. 1D, a high/preferential expression of *PTX3* occurs in the tumor cell subpopulations (CC_TNBC) characterized by the expression of *Keratin 8* (*KRT8*) (Figure S1A) and high genomic instability (Figure S1B). Conversely, low levels of *PTX3* transcript are observed in tumor stroma/microenvironment associated cells, including endothelial cells (Endo), cancer-associated fibroblasts (CAF), macrophages, dendritic cells and lymphocytes (T and NK cells) (Fig. 1D and S1). These data were strongly supported by RNAscope analyses performed on other samples from triple negative tumor biopsies showing high levels of PTX3 mRNA expression in tumor cells (pan CK^+^ cells), but not in stromal cells (Fig. 1E). These findings point to a central role of tumor cells in the expression and secretion of PTX3 in TNBC.

In keeping with data obtained from patient-derived samples of different BC subtypes and the finding that tumor cells are the main source of PTX3, analysis in the GOBO database revealed that the majority of TNBC cell lines express higher levels of *PTX3* mRNA in respect to the HER^+^ and ER^+^/PR^+^ counterpart (Fig. 1F). This was confirmed also by analyzing data about *PTX3* mRNA levels in BC cell lines reported in the EMBL-EBI expression atlas database (Fig. 1G) [31]. Accordingly, gene expression, Western blot and ELISA analyses (Fig. 1H) showed higher PTX3 expression and secretion in TNBC cell lines when compared to ER^+^/PR^+^ and HER2^+^ breast cancer cells.

### PTX3 modulation impacts on the aggressiveness of TNBC cells

**Fig. 2:**
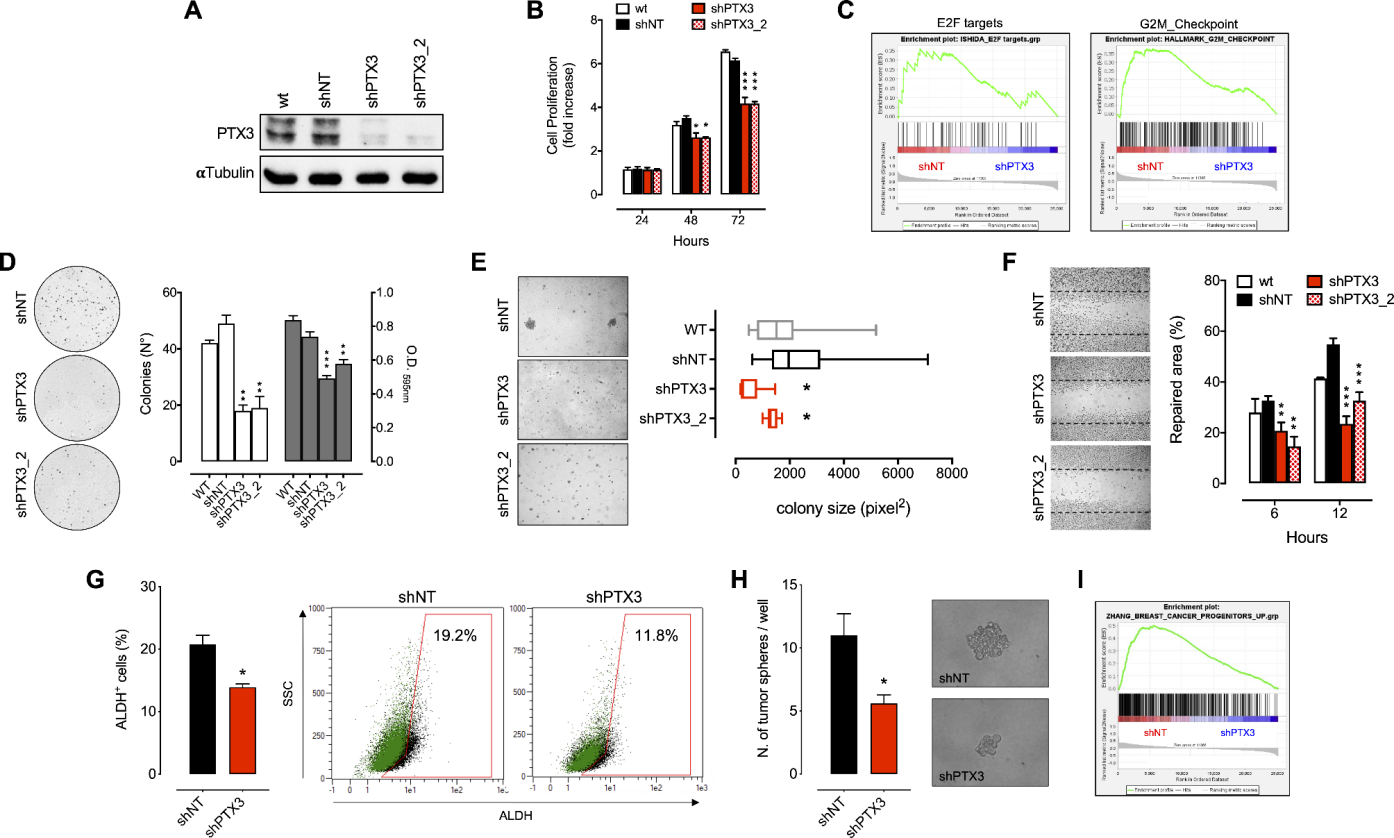
Effects of PTX3 silencing in MDA-MB-231 cells. (**A**) Western blot analysis of PTX3 silencing. (**B**) Cell proliferation assay by viable cell counting through cytofluorimetric analysis. (**C**) Gene set enrichment analysis (GSEA) of genes associated with proliferation. (**D**) Colony formation assay. White bars indicate the number of colonies, grey bars indicate the absorbance after crystal violet staining and solubilization of the colonies. (**E**) Soft agar assay. (**F**) Wound healing assay. (**G**) Percentage of ALDH-positive cells quantified by cytofluorimetric analysis. Green dots represent DEAB treated control cell population, black dots represent cell population not treated with DEAB. Gates have been set according to DEAB treated cell population. (**H**) Tumor sphere formation assay. (**I**) GSEA of genes associated with BC progenitors Data are the mean ± SEM, experiments were performed in triplicate. In box and whiskers graphs, boxes extend from the 25th to the 75th percentiles, lines indicate the median values, and whiskers indicate the range of values. *p < 0.05, **p < 0.01, ***p < 0.001

Based on the *in silico* and Western blot/ELISA data (Fig. 1F-H), representative human TNBC MDA-MB-231 (Fig. 2) and BT549 (Figure S2) cells expressing high levels of PTX3 were engineered to down-modulate *PTX3* expression. Transduction with two independent short-hairpins RNA (shRNA) efficiently down-modulated *PTX3* expression in both MDA-MB-231 and BT549 cells when compared to control (shNT) and wild type (wt) cells (Fig. 2A and S2A). In vitro characterization revealed that PTX3 silencing significantly reduced the proliferative capacity of shPTX3 cells in respect to control/shNT and wt cells (Fig. 2B and S2B). Accordingly, comparative Gene Set Enrichment Analysis (GSEA) performed on MDA-MB-231 cells revealed a significant downregulation of genes involved in cell proliferation and cycling, such as the E2F target genes, and genes involved in the G2-M phase transition (Fig. 2C). Also, shPTX3 cells showed a reduced clonogenic potential (Fig. 2D and S2C) as well as an impaired anchorage-independent growth capacity when seeded in soft agar (Fig. 2E and S2D). Moreover, shPTX3 cells were characterized by a reduced motility, as assessed in a wound repair assay (Fig. 2F and S2E).

Recently, it has been reported that PTX3 expression might confer stem-like traits to TNBC [21]. Here, we observed that after *PTX3* downregulation in MDA-MB-231 cells the percentage of ALDH^+^ cells (a functional marker of stem-like cell populations) (Fig. 2G) was significantly reduced, as well as the number and dimensions of tumor-spheres formed (Fig. 2H). Accordingly, GSEA revealed a significant downregulation of genes associated with BC progenitors and of mammary stem cells genes in MDA-MB-231 shPTX3 when compared to shNT cells (Fig. 2I and Figure S3A), and a low consensus stemness ranking (CSR) signature (CSR score) [32] was observed indicating a reduction in cancer stem cell content (Figure S3B). In keeping with previous reports [22], the reduced stem-like features that occur in MDA-MB-231 shPTX3 cells go along with a reduced phosphorylation of JNK and c-Jun (Figure S4A), and a subsequent significant downregulation of JNK target genes (Figure S4B).

**Fig. 3:**
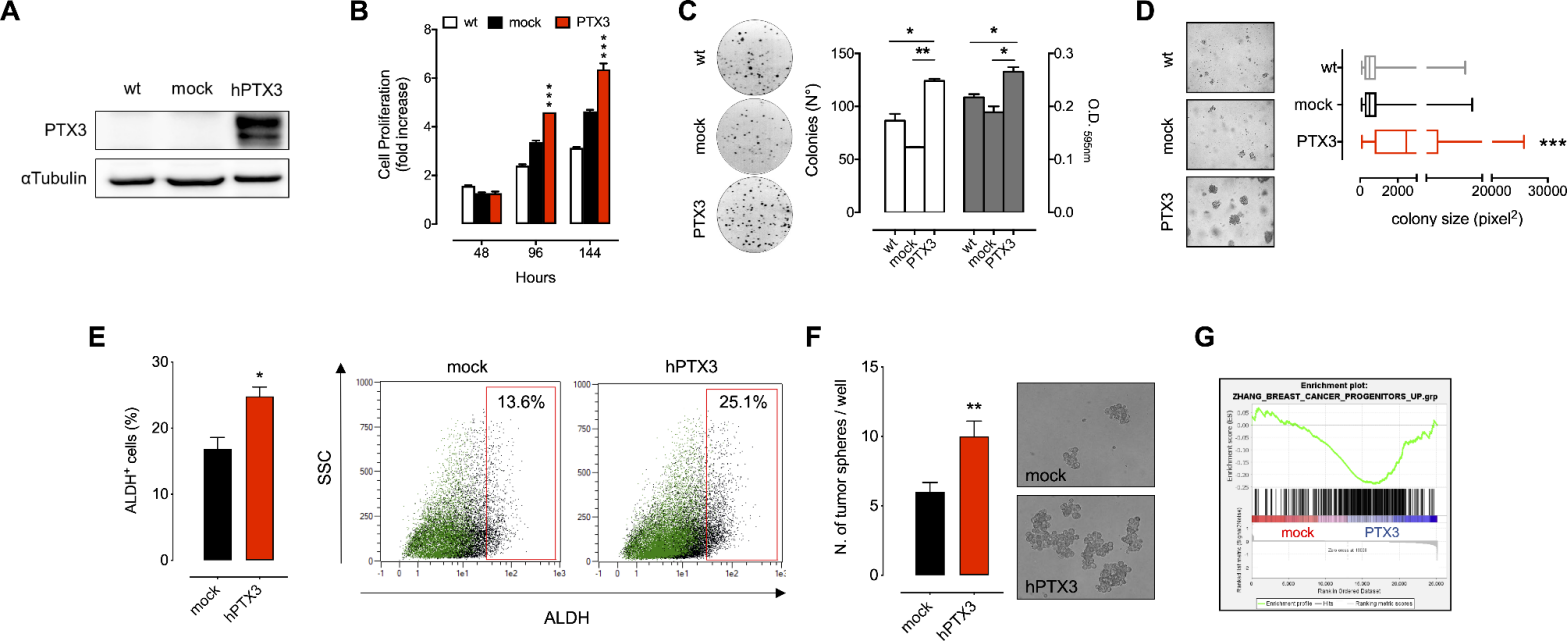
Effects of PTX3 overexpression in MDA-MB-468 cells. (**A**) Western blot analysis of PTX3 overexpression. (**B**) Cell proliferation assay by viable cell counting through cytofluorimetric analysis. (**C**) Colony formation assay. White bars indicate the number of colonies, grey bars indicate the absorbance after crystal violet staining and solubilization of the colonies (**D**) Soft agar assay. (**E**) Percentage of ALDH-positive cells quantified by cytofluorimetric analysis. Green dots represent DEAB treated control cell population, black dots represent cell population not treated with DEAB. Gates have been set according to DEAB treated cell population. (**F**) Tumor sphere formation assay. (**G**) GSEA of genes associated with BC progenitors Data are the mean ± SEM, experiments were performed in triplicate. In box and whiskers graphs, boxes extend from the 25th to the 75th percentiles, lines indicate the median values, and whiskers indicate the range of values. *p < 0.05, **p < 0.01, ***p < 0.001

Finally, in order to validate the results obtained following *PTX3* down-modulation, we generated stable *PTX3* overexpressing MDA-MB-468 cells (Fig. 3A). When compared to MDA-MB-231 cells, TNBC MDA-MB-468 cells are characterized by very low levels of PTX3 (Fig. 1G-H). As anticipated, *PTX3* overexpression in MDA-MB-468 cells resulted in increased cell proliferation (Fig. 3B), clonogenic potential (Fig. 3C) and capacity to growth under anchorage independent conditions (Fig. 3D). Also, the overexpression of *PTX3* significantly increased the percentage of ALDH^+^ cells (Fig. 3E) and the capacity of MDA-MB-468 PTX3 cells to form tumor-spheres (Fig. 3F). These augmented stem-like features in MDA-MB-468 PTX3 cells were further confirmed by a significant increase in the expression of genes associated with BC progenitors and mammary stem cells as assessed by GSEA (Fig. 3G and Figure S3A), and an increased CSR score (Figure S3B) [32]. Finally, in MDA-MB-468 cells overexpressing PTX3 increased phosphorylation levels of JNK and c-Jun were observed (Figure S4A) as well as a significant upregulation of JNK target genes (Figure S4B).

Altogether these data indicate that PTX3 strongly promotes TNBC cell growth. Indeed, in accordance with the correlation that occurs between *PTX3* expression and tumor aggressiveness in BC patients (Fig. 1C), high *PTX3* expressing MDA-MB-231 shNT and MDA-MB-468 PTX3 cells displayed a gene profile associated with high grade/more aggressive BC when compared to low *PTX3* expressing MDA-MB-231 shPTX3 and MDA-MB-468 mock cells, respectively (Figure S5).

### PTX3 modulation determines TNBC cell growth in vivo

**Fig. 4:**
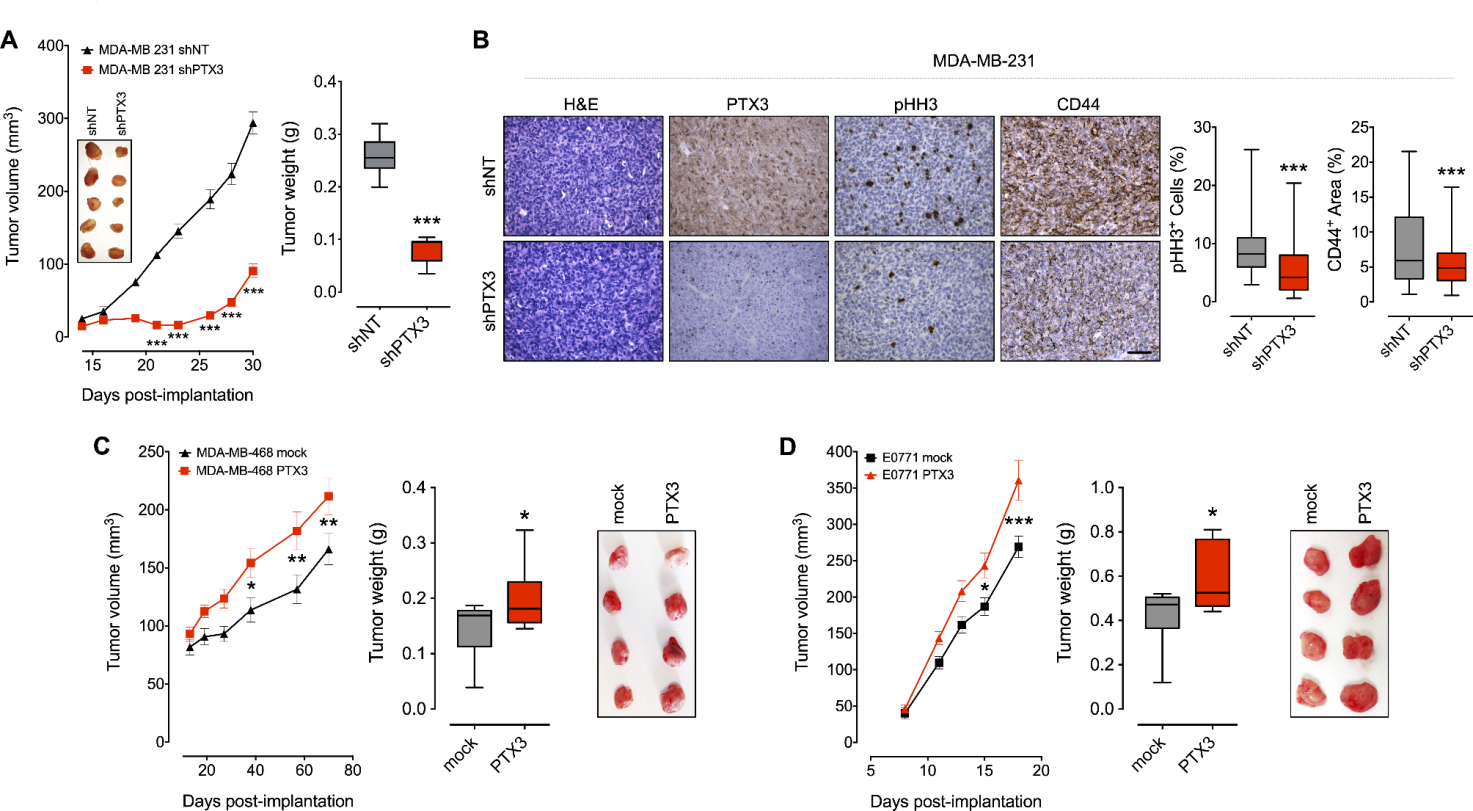
PTX3 affects TNBC cell growth in vivo. (**A**) Tumor growth and representative pictures (left panel) and weight (right panel) of control (shNT) and *PTX3*-silenced (shPTX3) MDA-MB-231 orthotopic tumors grown in immune-compromised mice. (**B**) Immunohistochemical analysis (left panel) of MDA-MB-231 shNT and shPTX3 tumors and quantification (right panel) of pHH3^+^ cells and CD44^+^ area by ImageJ analysis. Scale bar: 50 μm. (**C**) Tumor growth, weight and representative pictures of control (mock) and *PTX3*-overexpressing (PTX3) MDA-MB 468 orthotopic tumors grown in immune-compromised mice, and (**D**) of *PTX3*-overexpressing (PTX3) E0771 orthotopic tumors grown in immune competent syngeneic mice. In box and whiskers graphs, boxes extend from the 25th to the 75th percentiles, lines indicate the median values, and whiskers indicate the range of values. *n* = 8/10 mice/group; *p < 0.05, **p < 0.01, ***p < 0.001

The impact exerted by PTX3 modulation in TNBC cells was further assessed in vivo by orthotopic tumor models in immune-compromised mice. To this purpose, MDA-MB-231 shPTX3 and MDA-MB-231 shNT cells were grafted into the mammary fat pad and tumor growth was monitored. When compared to the corresponding controls, *PTX3* downregulation resulted in a significant decrease of MDA-MB-231 shPTX3 tumor growth (Fig. 4A). Accordingly, immunohistochemical analysis of representative MDA-MB-231 shPTX3 tumor samples showed reduced tumor cell proliferation (assessed by immunostaining for the pHH3 marker) when compared to MDA-MB-231 shNT lesions (Fig. 4B). Also, *PTX3* silenced tumors were characterized by a significant decrease of the stemness marker CD44 as assessed by immunostaining (Fig. 4B). Conversely, grafting of *PTX3* overexpressing MDA-MB-468 PTX3 cells into the mammary fat pad of immune-compromised mice resulted in an increased tumor burden in respect to mock lesions (Fig. 4C).

To confirm these findings in immune competent syngeneic mice, we generated *PTX3* overexpressing murine TNBC E0771 cells (E0771 PTX3 cells) (Figure S6A). Again, PTX3 overexpression conferred an increased proliferative rate (Figure S6B) and capacity to growth under anchorage independent conditions (Figure S6C) in vitro, as well as higher tumor growth capacity to E0771 PTX3 cells when implanted orthotopically into syngeneic C57BL/6 female mice (Fig. 4D).

Together, these data indicate that the modulation of PTX3 levels in TNBC cells exerts a significant impact on tumor growth in vivo, high levels of PTX3 being associated with an elevated tumorigenic potential, as observed in human MDA-MB-231 shNT and MDA-MB-468 PTX3 tumors as well as in murine E0771 PTX3 tumors, whereas reduced levels of the protein confer a less aggressive phenotype, as observed in MDA-MB-231 shPTX3, MDA-MB-468 mock and E0771 mock tumors.

### PTX3 activates TLR4/IRAK1 signaling in TNBC cells

**Fig. 5:**
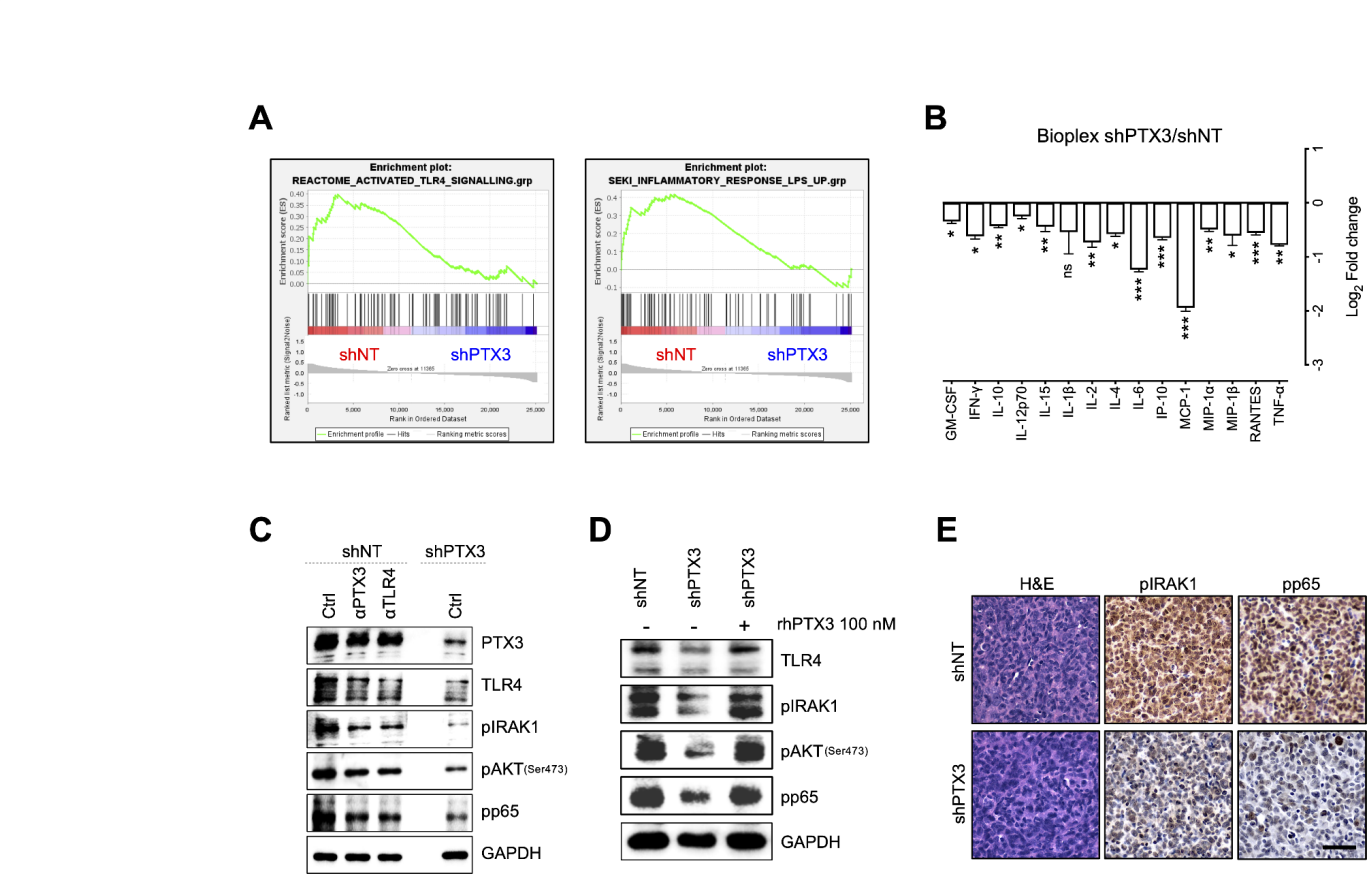
PTX3 activates TLR4/IRAK1 signaling in TNBC. (**A**) GSEA of MDA-MB-231 (shNT vs. shPTX3) genes associated with TLR4 signaling (left panel) and inflammatory response to LPS (right panel). (**B**) Bio-Plex assay of secreted pro-inflammatory cytokines from MDA-MB-231 cells (shPTX3 vs. shNT). (**C**) Western blot analysis of MDA-MB-231 shNT and shPTX3 cells treated or not with 10 µg/ml of anti-PTX3 or anti-TLR4 antibodies for 6 h. (**D**) Western blot analysis of MDA-MB-231 shNT and shPTX3 cells treated or not with 100 nM of recombinant human PTX3 (rhPTX3) for 24 h. (**E**) Immunohistochemical analysis of MDA-MB-231 shNT and shPTX3 tumors. Scale bar: 50 μm Data are the mean ± SEM, experiments were performed in triplicate. *p < 0.05, **p < 0.01, ***p < 0.001

To get insights about the molecular mechanism(s) by which PTX3 promotes TNBC, the activation of cell surface receptor-mediated pathways was investigated through GSEA using gene expression profiling data. This analysis revealed a significant correlation between TLR4 signaling and *PTX3* expression in MDA-MB-231 cells, TLR4 signaling being activated when *PTX3* is expressed and inactivated when *PTX3* is silenced (Fig. 5A). In keeping with the reduced activation of the TLR4 pathway in low expressing *PTX3* cells, GSEA showed a significant decrease in the expression of genes related to the LPS-mediated inflammatory response in MDA-MB-231 shPTX3 compared to MDA-MB-231 shNT cells (Fig. 5A). Indeed, Ingenuity Pathway Analysis (IPA) of differentially expressed genes revealed the presence of a significant link between PTX3 and Akt/NF-ĸB pathways (Figure S7A), together with a significant downregulation of NF-ĸB target genes (Figure S7B). Accordingly, Bio-Plex assay revealed a reduced amount of secreted pro-inflammatory cytokines in *PTX3*-silenced cells (Fig. 5B).

In keeping with these data, Western blot analysis revealed a strong activation of TLR4 signaling in *PTX3* expressing cells, as indicated by high levels of TLR4 expression and the activation/phosphorylation of the downstream IRAK1, Akt and NF-kB (p65) mediators in MDA-MB-231 shNT when compared to MDA-MB-231 shPTX3 (Fig. 5C). Accordingly, TLR4 signaling resulted significantly increased in MDA-MB-468 PTX3 cells when compared to MDA-MB-468 mock cells (Figure S8A). Of note, the blockade of PTX3 or TLR4 using both an anti-PTX3 or an anti-TLR4 antibody significantly down-modulated TLR4 signaling in MDA-MB-231 shNT cells, reaching levels similar to those observed in MDA-MB-231 shPTX3 cells (Fig. 5C). Also, as a proof of concept, treatment with exogenous recombinant PTX3 protein restored TLR4 signaling activation in MDA-MB-231 shPTX3 cells (Fig. 5D). Finally, the strong correlation between *PTX3* expression and TLR4 signaling activation was confirmed in vivo where MDA-MB-231 shPTX3 tumors showed reduced levels of IRAK1 and NF-kB (p65) phosphorylation compared to MDA-MB-231 shNT tumors (Fig. 5E).

Altogether these results indicate that *PTX3* expression induces the activation of the TRL4/IRAK1/NF-kB pathway known to play a relevant role in mediating TNBC aggressiveness [23, 24].

### TLR4 blockade impairs the tumorigenic activity of PTX3 in TNBC

**Fig. 6:**
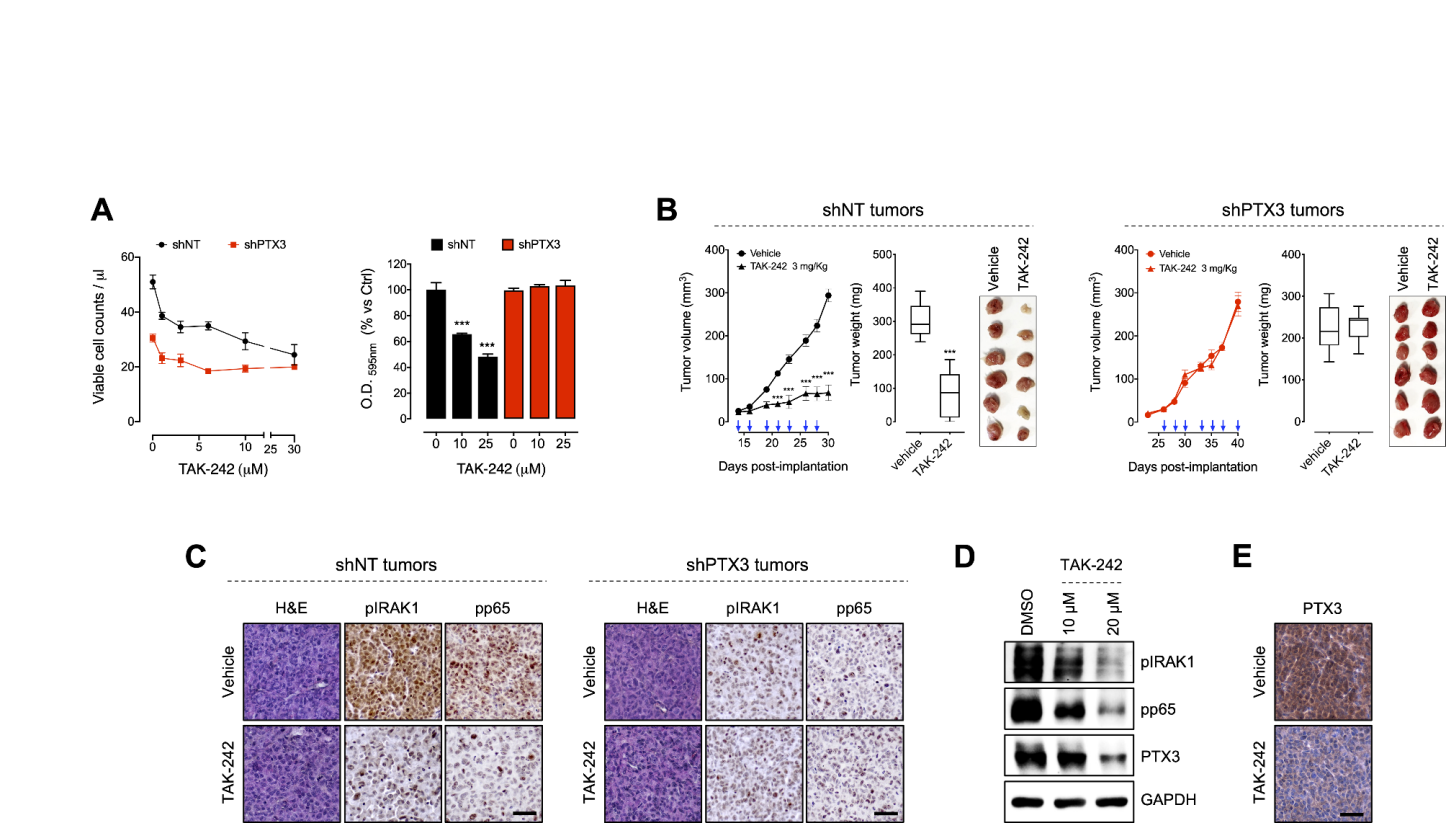
TLR4 inhibition hampers PTX3 tumorigenic activity in TNBC. (**A**) Left panel: inhibition of MDA-MB-231 shNT and shPTX3 cell proliferation after treatment with increasing doses of TAK-242. Right panel: colony formation assay of MDA-MB-231 shNT and shPTX3 cells treated or not with TAK-242. (**B**) Tumor growth and weight of MDA-MB-231 shNT (left panel) and shPTX3 (right panel) orthotopic tumors implanted into immune-compromised mice and treated or not with 3 mg/Kg TAK-242. Blue arrows indicate the days of treatment. *n* = 8/10 mice/group. (**C**) Immunohistochemical analysis of MDA-MB-231 shNT (left panel) and shPTX3 (right panel) tumors treated or not with 3 mg/Kg TAK-242 (same tumors shown in **B**). Scale bar: 50 μm. (**D**) Western blot analysis of MDA-MB-231 shNT cells treated or not with 10 or 20 µM of TAK-242. (**E**) Immunohistochemical analysis for PTX3 expression of MDA-MB-231 shNT tumors treated or not with 3 mg/Kg TAK-242 (same tumors shown in **B**). Scale bar: 50 μm Data are the mean ± SEM, experiments were performed in triplicate. In box and whiskers graphs, boxes extend from the 25th to the 75th percentiles, lines indicate the median values, and whiskers indicate the range of values. *n* = 8/10 mice/group; ***p < 0.001

To further assess the role of PTX3-mediated TLR4 activation in promoting TNBC, we investigated the antitumor activity of the TLR4 inhibitor TAK-242 in MDA-MB-231 shNT and shPTX3 cells. In vitro, TAK-242 significantly reduced the proliferation and the clonogenic capacity of MDA-MB-231 shNT cells, but not of MDA-MB-231 shPTX3 cells (Fig. 6A). Similar results were obtained with BT549 shNT and shPTX3 cells (Figure S8B). Accordingly, TAK-242 treatment impaired the clonogenic capacity of MDA-MB-468 PTX3 cells, but not of MDA-MB-468 mock cells (Figure S8C). These data suggest that TLR4 inhibition may affect only TNBC cells expressing high levels of PTX3.

To confirm these findings, the antitumor activity of TAK-242 was assessed in vivo in MDA-MB-231 and MDA-MB-468 orthotopic tumors. Again, TLR4 inhibition by TAK-242 treatment significantly impaired the growth of PTX3-expressing tumors (MDA-MB-231 shNT and MDA-MB-468 PTX3), but did not affect the growth of tumors not expressing PTX3 (MDA-MB-231 shPTX3 and MDA-MB-468 mock) (Fig. 6B and Figure S9). This effect was paralleled by a strong reduction of IRAK1 and NF-kB (p65) phosphorylation in MDA-MB-231 shNT tumors treated with TAK-242 compared to vehicle-treated tumors (Fig. 6C). In contrast, TAK-242 treatment did not affect IRAK1 and NF-kB (p65) phosphorylation in MDA-MB-231 shPTX3 tumors (Fig. 6C) that are characterized by a reduced activation of the TRL4 pathway (Fig. 5E).

Finally, in order to investigate if a PTX3/TLR4 autocrine loop of stimulation exists in TNBC cells, the expression of PTX3 in MDA-MB-231 shNT cells and tumor xenografts was assessed after TLR4 inhibition. Interestingly, the blockade of TLR4 signaling by the TLR4 inhibitor TAK-242 significantly reduced the expression of PTX3 both in vitro (Fig. 6D) and in vivo (Fig. 6E), suggesting that PTX3 expression in TNBC cells is under the control of TLR4 signaling activation. These findings are in keeping with data reported in the literature showing that PTX3 expression is controlled by NF-kB activation in basal-like breast cancers [21]. Indeed, reduced levels of PTX3 were paralleled by the inactivation of NF-kB (p65) after TAK-242 treatment (Fig. 6C-D), indicating that PTX3 regulates its own expression by TLR-4 mediated NF-kB activation.

Altogether these results strongly indicate that PTX3 produced by tumor cells exerts its tumorigenic activity in TNBC via TLR4 activation which in turn regulate PTX3 expression through NF-kB activation, thus generating a PTX3/TLR4 autocrine loop of stimulation in tumor cells that fosters TNBC growth and progression.

## Discussion

In the last decades, considerable progresses have been made in BC treatment, especially through the introduction of targeted therapies against the signaling pathways governing cancer onset and progression [33–35]. For instance, ER, PR and HER2 play key roles in the evolution of the majority of BCs, and selective targeting of these proteins has enabled the inhibition of their associated pathways, leading to a better prognosis for tumors that are positive for these receptors [36–38]. At variance, TNBC, that accounts for 10–15% of all BCs, lacks of effective specific targeted therapies due to its aggressive clinical behavior and displays a risk death of 70% in the five years following diagnosis [39–41]. For these reasons, the characterization of new molecular pathways and/or alternative druggable targets is of great interest for TNBC [42].

PTX3 may exert anti-tumor or pro-tumor effects depending on tumor type and context [43, 44]. In this frame, limited experimental evidence suggests that PTX3 may be endowed with a tumor-promoting activity in TNBC. Indeed, PTX3 has been shown to be a marker of poor prognosis in TNBC patients [21]. However, the cellular source of PTX3, its biological effects and the mechanism(s) by which PTX3 exerts its pro-tumorigenic activity in TNBC have not been investigated so far.

Here we show that (i) among all subtypes of BC, PTX3 is highly expressed in the most aggressive TNBC subtype, (ii) the main source of PTX3 in TNBC patient-derived samples is represented by tumor cells rather than the stromal/immune component, and (iii) *PTX3* expression by tumor cells fosters the tumorigenic potential of TNBC by activating a PTX3/TLR4 autocrine loop.

These findings indicate that PTX3 produced and secreted by tumor cells may act as an autocrine factor able to condition TNBC cell behavior. Relevant to this point, our data show for the first time that PTX3 exerts its pro-tumor activity in TNBC by activating TLR4/IRAK1/NF-kB signaling in tumor cells. Indeed, GSEA, Western blot and immunohistochemical analyses demonstrate that the TLR4 pathway is activated when *PTX3* is expressed and inactivated when *PTX3* is silenced in both in vitro and in vivo TNBC models. The strict correlation between *PTX3* expression and TLR4 activation was confirmed by the fact that TLR4 blockade impairs PTX3-mediated tumorigenic activity in vitro and in vivo. Also, exogenous PTX3 is able to restore the activation of TLR4 pathway in *PTX3* silenced TNBC cells.

PTX3 has been shown to exert a protective antifungal activity by directly activating TLR4 through the binding to myeloid differentiation protein 2 (MD-2), an accessory protein of TLR4 [45]. In cancer, a PTX3/TLR4 interaction has been recently reported only for invasive melanoma [46]. Our data extend these observations and strongly indicate that the PTX3/TLR4 system may play a non-redundant role in TNBC aggressiveness. Accordingly, TLR4 has been shown to be upregulated in human BC tissues [47, 48] and constitutive activation of IRAK1 and NF-kB, key downstream effectors of TLR4 signaling, has been frequently reported in TNBC [49–51]. The activation of this key pathway leads to the expression of pro-inflammatory cytokines and anti-apoptotic genes that foster aggressive growth, stemness and chemoresistance in TNBC cells. Indeed, pharmacological inhibition of TLR4 or IRAK1 has been reported to abolish the growth and metastatic progression of TNBC [48, 49]. In this frame, our data indicate that PTX3 secreted by tumor cells promotes the activation of the TLR4/IRAK1 pathway in TNBC cells, and that the expression of PTX3 itself may determine the antitumor responses to TLR4 inhibition. In fact, TLR4 inhibition by TAK-242 treatment significantly impaired the proliferation and the clonogenic capacity in vitro and tumorigenic activity in vivo of *PTX3*-expressing TNBC cells but did not affect the tumorigenic potential of *PTX3*-silenced cells.

In a therapeutic perspective, our data indicate that the PTX3/TLR4 autocrine loop may represent a novel therapeutic target for TNBC. So far, several TLR antagonists/inhibitors have been investigated in clinical trials for the therapy of inflammatory diseases and disorders of the vascular system [52]. In this frame, our observations suggest that the direct targeting of PTX3 or TLR4 may represent a promising therapeutic approach for the treatment of TNBC where TLR4 signaling activation strictly depends on PTX3 expression. This implies that TLR4 inhibition may affect only those TNBC lesions that express high levels of PTX3, a criterium to be taken into account for the selection of patients undergoing future anti-TLR4 therapies in TNBC. On the other side, our findings reinforce the therapeutic significance of recent approaches under phase II/III clinical evaluation [42, 53] based on targeting TLR4 downstream effectors, such as Akt and NF-kB, in TNBC patients.

## Conclusions

In conclusion, we demonstrated that PTX3 is highly expressed in the most aggressive TNBC subtype and that *PTX3* expression by tumor cells fosters the tumorigenic potential of TNBC. Of note, our data revealed that the PTX3/TLR4 autocrine loop sustains TNBC growth and aggressiveness and determines the antitumor efficacy of TLR4 inhibition in TNBC. Altogether, our findings suggest that the direct targeting of PTX3 or TLR4 may represent a promising novel therapeutic approach for the treatment of TNBC as well as other tumor types where TLR4 signaling activation strictly depends on PTX3 expression.

### Electronic supplementary material

Below is the link to the electronic supplementary material.

Supplementary Material 1

## Data Availability

Further information and requests for reagents and resources should be directed to and will be made available by the corresponding authors upon reasonable request. The datasets generated and analyzed during the current study are available in the GEO repository (code GSE188315).

## Abbreviations

TLR4: Toll Like Receptor 4
PTX3: Pattern recognition receptor long pentraxin-3
TNBC: Triple negative breast cancer
GOBO: Gene Expression-Based Outcome for Breast Cancer Online
BC: Breast cancer
ER: Estrogen receptor
PR: Progesterone receptor
HER2: Human epidermal growth factor receptor 2
FGF: Fibroblast growth factor
EMT: Epithelial-to-mesenchymal transition
ALDH: Aldehyde dehydrogenase
DEAB: Diethylaminobenzaldehyde
HR: Hormone receptor
IPA: Ingenuity Pathway Analysis
GSEA: Gene Set Enrichment Analysis
GEP: Gene Expression Profiling
MD-2: Myeloid differentiation protein 2
