# Supplementary material for: The PTX3/TLR4 autocrine loop as a novel therapeutic target in triple negative breast cancer
Source: Exp Hematol Oncol. 2023 Sep 25;12:82. doi: 10.1186/s40164-023-00441-y (PMC10519006; doi:10.1186/s40164-023-00441-y)
Supplement: Supplementary file 1 — Supplementary Material 1 [file 40164_2023_441_MOESM1_ESM.pdf]

SUPPLEMENTARY DATA

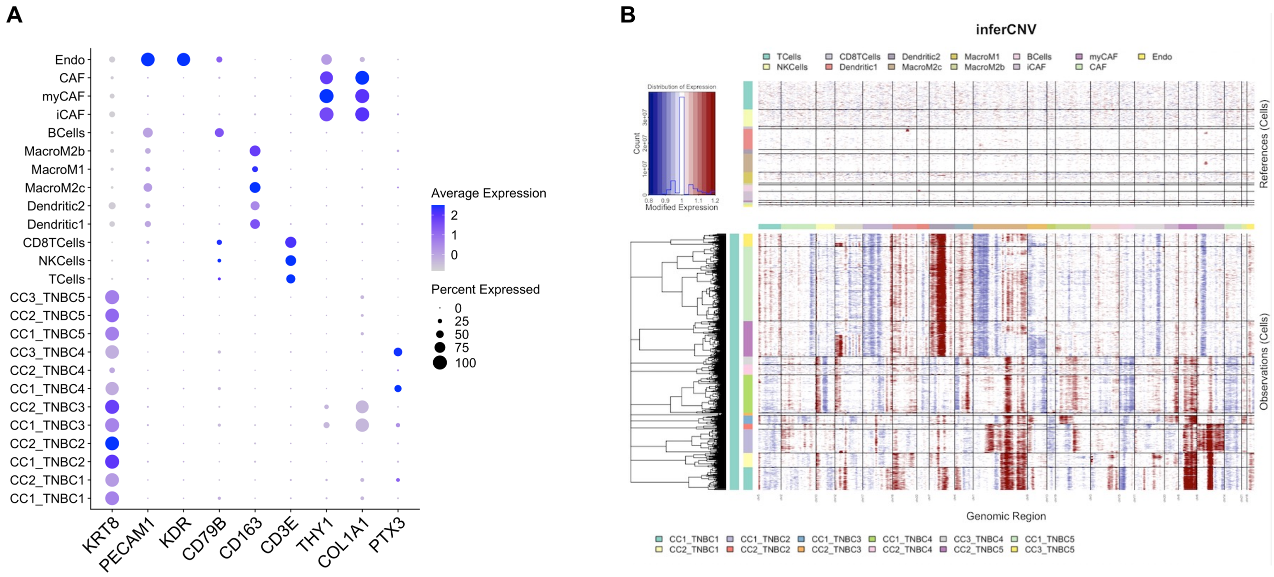

Figure S1. Single cell analysis of TNBC samples. (**A**) For each cell population (y-axis), gene expression levels of cell-specific markers (x-axis) is shown. (**B**) Genomic instability by copy number variation (CNV) analysis for each cell population confirming the presence of genomic abnormalities in tumor cells compared to stromal and immune cell population.

Same dataset as the one used in the Figure 1D.

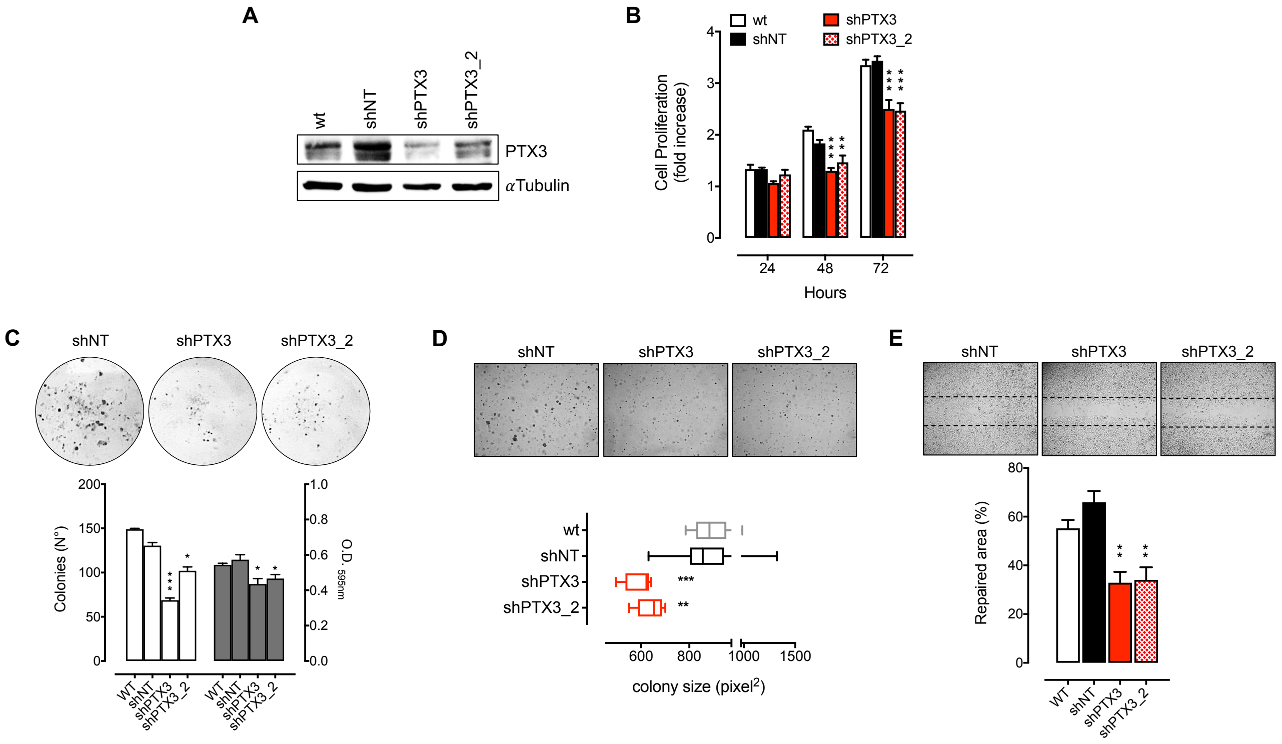

Figure S2. PTX3 silencing affects the biological features of BT549 TNBC cell line. (**A**) Western blot analysis of PTX3 silencing. (**B**) Cell proliferation assay by viable cell counting through cytofluorimetric analysis. (**C**) Colony formation assay. White bars indicate the number of colonies, grey bars indicate the absorbance after crystal violet staining and solubilization of the colonies. (**D**) Soft agar assay. (**E**) Wound healing assay. Data are the mean ± SEM, experiments were performed in triplicate. In box and whiskers graphs, boxes extend from the 25th to the 75th percentiles, lines indicate the median values, and whiskers indicate the range of values. *$p < 0.05$, **$p < 0.01$, ***$p <$ 0.001.

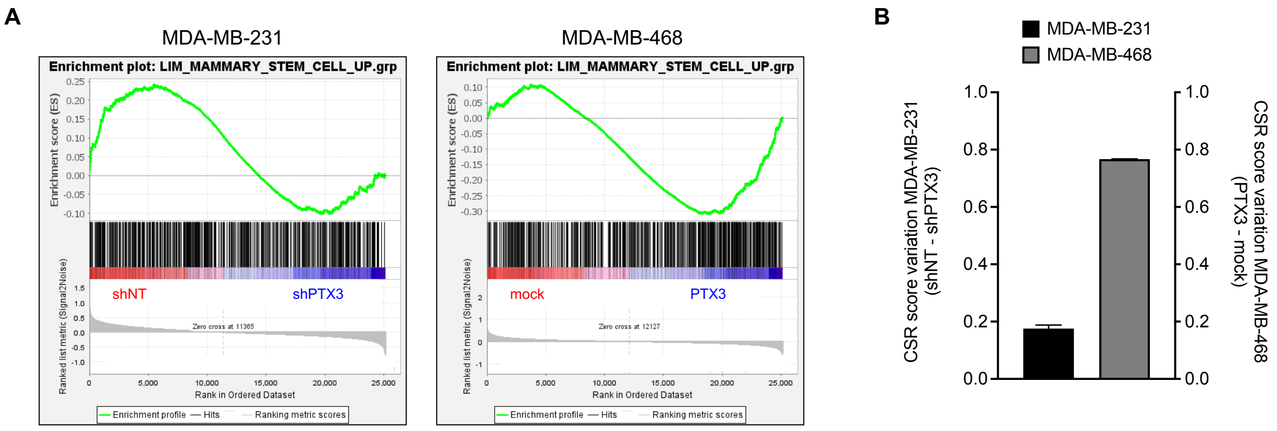

Figure S3. PTX3 modulation affects TNBC cell stemness. A) GSEA for stem-related genes. **B)** Consensus Stemness Ranking **(**CSR) score variation for MDA-MB-231 (shNT - shPTX3) and MDA-MB-468 (PTX3 - mock). Matched gene expression to coefficient provided in [32] using Gene Symbol. We found 83 genes out of 100 provided in the CSR signature. Duplicated coefficients in signature were averaged. Computed linear combination of each gene expression based on the signature coefficients (excluding the intercept). We modelled the CSR score using a robust linear model to test the “high PTX” vs “low PTX” contrast within cell type [32].

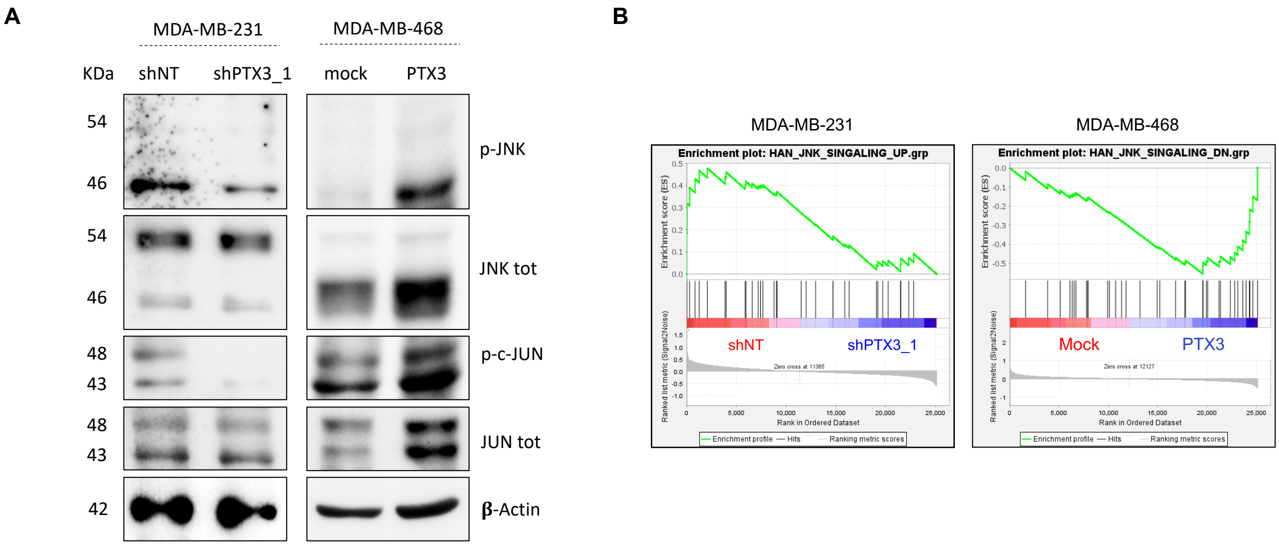

Figure S4. PTX3 silencing inactivates JNK signaling in MDA-MB-231 cells whereas PTX3 overexpression activates JNK signaling in MDA-MB-468 cells. (**A**) Western blot analysis for JNK and c-Jun phosphorylated levels. (**B**) GSEA of genes associated with JNK signaling.

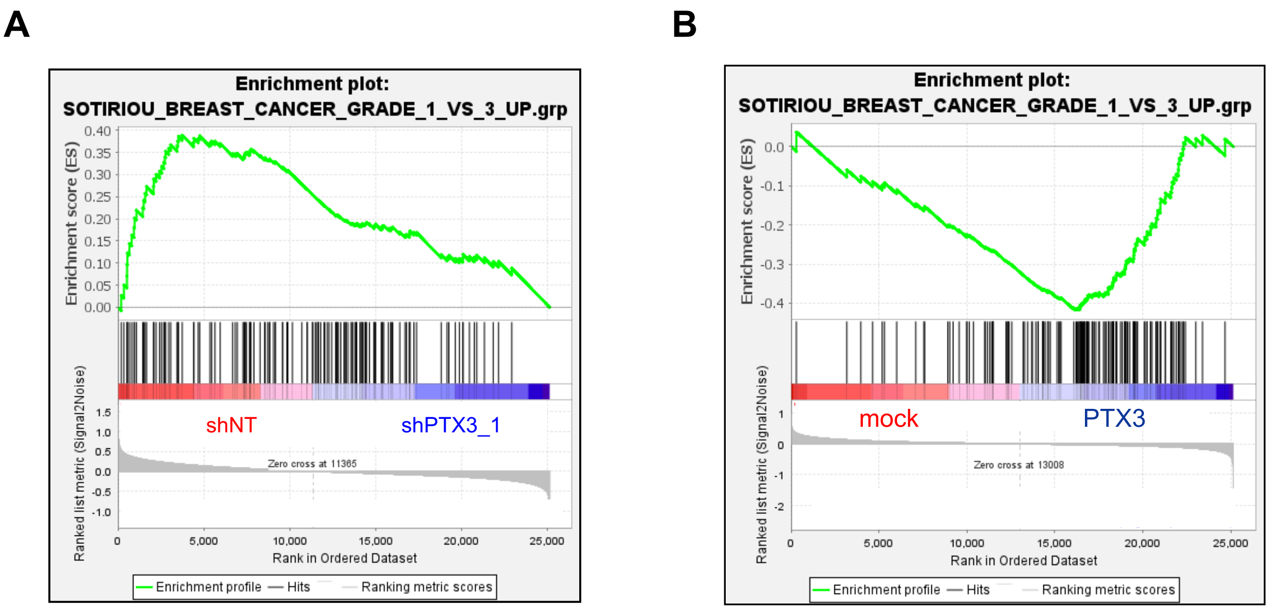

Figure S5. Correlation between PTX3 expression and BC grade. GSEA of genes associated with BC grade in MDA-MB-231 (**A**) and MDA-MB-468 (**B**) cells.

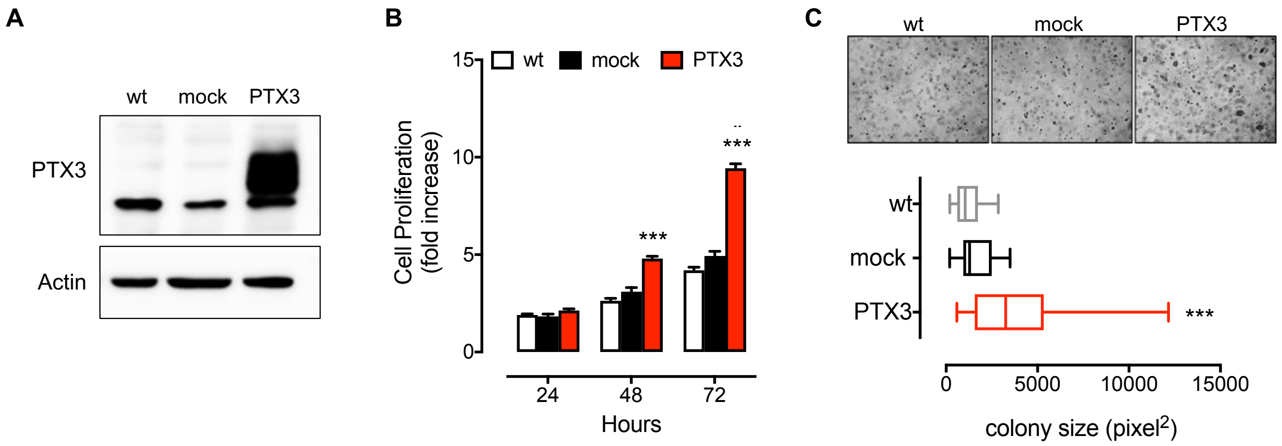

Figure S6. PTX3 overexpression in murine E0771 cells. *PTX3* overexpressing murine E0771 cells were generated and tested for (**A**) PTX3 expression by Western blot analysis, (**B**) proliferative capacity by viable cell counting and (**C**) anchorage-independent growth capacity by soft agar assay. Data are the mean ± SEM, experiments were performed in triplicate. In box and whiskers graphs, boxes extend from the 25th to the 75th percentiles, lines indicate the median values, and whiskers indicate the range of values. ***$p < 0.001$.

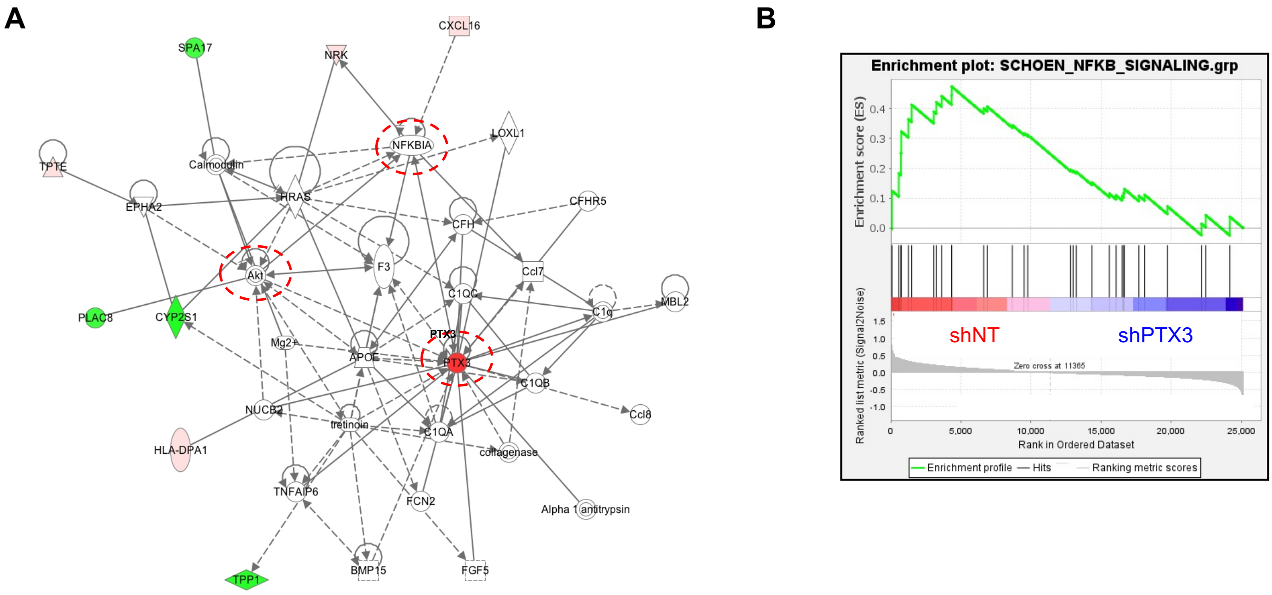

Figure S7. Link between PTX3 and Akt/NF-kB pathways. (**A**) Ingenuity Pathway Analysis (IPA) of differentially expressed genes obtained from gene expression profiling of MDA-MB-231 shNT *vs* shPTX3 cells. Node color indicates up-regulated genes (red) and down-regulated genes (green); edges (lines and arrows between nodes) represent direct (solid lines) and indirect (dashed lines) interactions between molecules as supported by information in the Ingenuity knowledge base. Node shapes represent functional classes of gene products. Red dashed circles indicate the signaling axis to which the majority of genes converge. (**B**) GSEA of genes associated with NF-kB signaling.

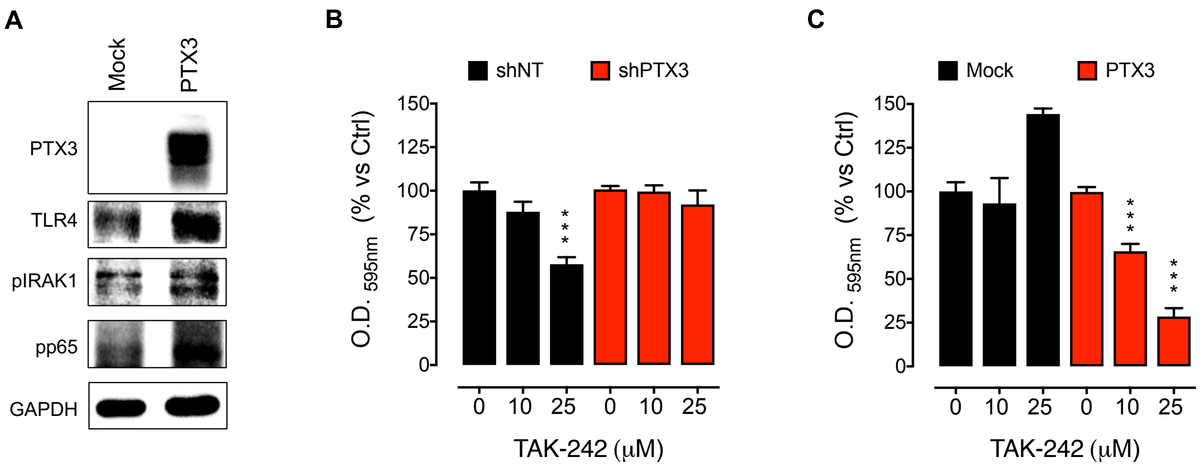

Figure S8. PTX3 modulation regulates TLR4 signaling activation and antitumor activity of TLR4 inhibitor TAK-242. (**A**) Western blot analysis of MDA-MB-468 mock and PTX3 overexpressing cells. (**B-C**) Clonogenic capacity of BT549 shNT and shPTX3 cells (**B**) and MDA-MB-468 mock and PTX3 cells (**C**) in presence or absence of 10 or 25 μM TAK-242. Data are the mean ± SEM, experiments were performed in triplicate. ***$p < 0.001$.

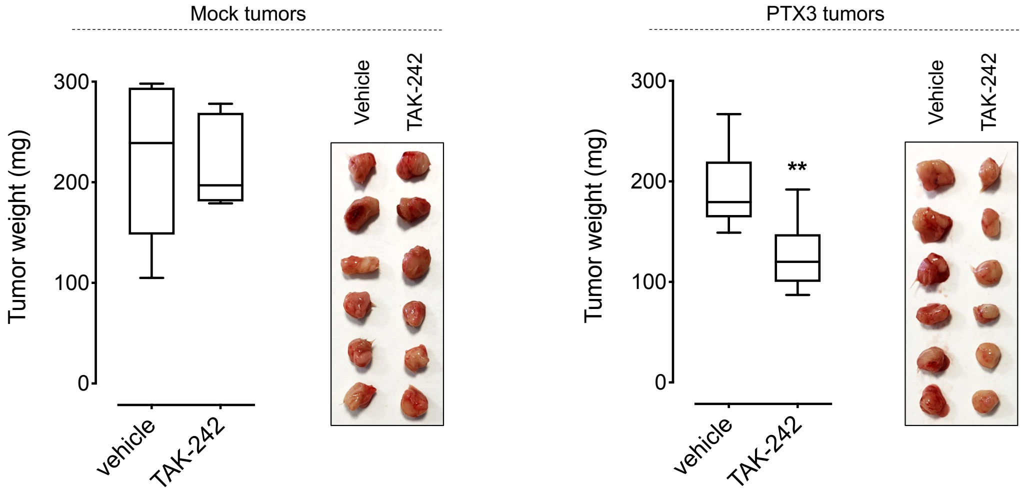

Figure S9. Tumor growth of MDA-MB-468 tumors treated with TAK-242. Weight of MDA-MB-468 mock (left panel) and PTX3 (right panel) orthotopic tumors implanted into immune-compromised mice and treated or not with 3 mg/Kg TAK-242. *n* = 8/10 mice/group.

In box and whiskers graphs, boxes extend from the 25th to the 75th percentiles, lines indicate the median values, and whiskers indicate the range of values. *n* = 8/10 mice/group; $^{**}p < 0.01$.

Sample N°	Age at diagnosis	Molecular type	Histological type	Tumor grade	Clinical Stage	ER	PR	Ki-67	erb-B2	FISH HER2	Metastasis	Survival (2023)
1	37	TN	invasive ductal carcinoma	Bloom 3	cT1cN0M0	negative	negative	50%	1+	negative	-	Alive
2	44	TN	invasive ductal carcinoma	Bloom 3	cT3N1M0	negative	negative	80%	negative	not tested	liver, lung, ganglion	dead age 45
3	51	TN	bifocal ductal carcinoma	Bloom 3	cT3N1M0	negative	negative	60%	1+	negative	-	Alive
4	78	TN	invasive ductal carcinoma	Bloom 3	cT2mN1M0	negative	negative	40%	1+	negative	brain	dead age 79
5	82	TN	invasive ductal carcinoma	Bloom 3	cT3N1M0	negative	negative	80%	negative	negative	ganglion, lung, bone	dead age 84
6	44	TN	invasive ductal carcinoma	Bloom 3	cT3N1M0	negative	negative	80%	negative	not tested	liver, lung, ganglion, bone	dead age 45
7	24	TN	bifocal ductal carcinoma	Bloom 3	cT1cN0M0	negative	negative	90%	negative	negative	-	Alive
8	46	ER+/PR+/HER+	invasive ductal carcinoma	Bloom 3	cT2N0M0	positive	positive	40%	2+	positive	-	Alive
9	46	ER+/PR+/HER+	invasive ductal carcinoma	Bloom 2	cT2N0M0	positive	positive	12%	2+	positive	-	Alive
10	50	ER+/PR+/HER+	invasive ductal carcinoma	Bloom 1	cT1cN0M0	positive	positive	10%	1+	not tested	-	Alive
11	73	ER+/PR+	invasive ductal carcinoma	Bloom 1	cT2N0M0	positive	positive	25%	1+	not tested	-	dead age 81
12	70	ER+/PR+	invasive ductal carcinoma	Bloom 3	cT3mN1M0	positive	positive	10%	negative	not tested	-	Alive
13	61	HER2+	invasive ductal carcinoma	Bloom 3	cT2N0M0	negative	negative	60%	2+	positive	-	Alive
14	85	HER2+	invasive ductal carcinoma	Bloom 3	cT2N0M0	negative	negative	60%	2+	positive	-	dead age 86
15	48	HER2+	invasive ductal carcinoma	Bloom 3	cT2N0M0	negative	negative	50%	2+	positive	-	Alive

Table S1. Clinical data of patients whose tumor samples have been used for Western blot analysis in Figure 1B.
